# Resetting the compass: exploring the implicit messages of orientation to a community-engaged medical school

**Published:** 2017-02-24

**Authors:** Rachel Ellaway, Tim Dubé, Gerry Cooper, Lisa Graves

**Affiliations:** 1 Cumming School of Medicine, University of Calgary, Calgary, Alberta, Canada; 2 Centre for Medical Education, McGill University, Montréal, Quebec, Canada; 3 Schulich School of Medicine & Dentistry, Western University, Windsor, Ontario, Canada; 4 Western Michigan University Homer Stryker MD School of Medicine, Kalamazoo, Michigan, US; 5 Northern Ontario School of Medicine, Sudbury, Ontario, Canada

## Abstract

**Background:**

Although students’ transition into medical school is a critical step in their professional journey, orientation has been relatively under-researched, particularly with regard to its intersections with schools’ social missions. This paper reports on a study looking at the implicit messages of orientation to the Northern Ontario School of Medicine’s undergraduate program.

**Methods:**

An extended mixed methods study was conducted to look at different aspects of the School’s Orientation Week. The term “hidden curriculum” was used to shape inquiry, both in its broad sense of implicit educational experiences and messages and in its more specific sense of the educational messages sent by a medical school’s culture and activities. Data were collected using participant surveys, focus groups, and interviews. Transcripts and free-text survey responses were analyzed to identify underlying themes.

**Results:**

Orientation Week was generally well received and was generally perceived by different stakeholders (such as students, school leaders, and community members) as a positive and necessary undertaking. However, there were points of contention and confusion that created a hidden curriculum with respect to participants’ identities, both as students and as future health professionals.

**Conclusion:**

Orientation to undergraduate medical training can be successfully linked to a school’s social mission, but in doing so it can send complex and unintended messages to the participants that may be perceived quite differently based on their circumstances and expectations.

## Introduction

Orientation takes place on the threshold of medical education; it marks the point at which a student joins a medical school and starts their professional education. Orientation differs by school, but it typically involves a mixture of practical and professional information, and social activities.^1^ Although this is a critical step in a student’s professional journey, there has been comparatively little research into the qualities of medical school orientation or into the ways in which it can be made more effective.^1^

There are many tacit messages, both intended and unintended, that students pick up from their studies. These are often collectively described as the “hidden curriculum.”^2^,^3^ There have been hidden curriculum issues associated with many aspects of medical education, and while some (such as conflicting role modeling) have received much attention,^4^ others, such as the initial orientation to medical training, have been less substantially researched.^1^

We developed an extended study to explore the nature and impact of undergraduate orientation practices at the Northern Ontario School of Medicine, a Canadian school with a strong social mission. This paper reports on one dimension of this larger study, a review of the hidden curriculum of orientation to the undergraduate medical program. Our findings are intended to inform the emerging discourse around medical school orientation and around transitions in medical education in general, to contribute to the literature on the hidden curriculum in medical education, and to provide guidance to the growing number of community-engaged medical schools on issues associated with orientation occurring within community contexts.

## Background

Orientation is generally intended to provide new medical students with sufficient knowledge of the realities and expectations of being in medical school to allow them to function effectively in their new role.^1^ Orientation typically involves a combination of professional, social, and practical experiences that are expressed in many different ways in many different contexts.^1^ Despite the critical importance of orientation, researchers have paid relatively little attention to the transition into medical school. When orientation has been explored, the focus has tended to be on its ceremonial and symbolic dimensions.^5^–^8^

Although orientation is a critical transition, the literature on transitions has tended to focus on transitions during medical education programs rather than on entry to them.^9^–^11^ The evidence we have suggests that transitions in general (such as from pre-clinical to clinical, from undergraduate to postgraduate training, or from residency to independent practice) can be particularly challenging as they can send mixed messages, create identity conflicts, and lead to fragmented learning experiences for students.^12^,^13^

Researchers have distinguished different interrelated concepts of what medical students learn including formal, informal, and hidden curricula. The concept of the “hidden curriculum” has been used to describe the tacit and mixed messages that educational programs send to their learners.^2^,^3^ Several authors have broadened our understanding to consider different kinds of messages including: a more specific concept of hidden curriculum (messages taken from the structure of a program or school);^14^ informal curriculum (messages taken from individual interactions with members of a school);^15^,^16^ null curriculum (that which is explicitly omitted from the formal curriculum);^17^ and rhetorical curriculum (that which comes from the pronouncements of authority figures).^18^ Hafferty and others have situated some of these concepts within medical education by focusing on the professionalism conflicts between the ideals and actualities of bedside teaching.^19^ These different kinds of non-formal curricula may have positive, as well as negative, consequences. Exploring them can help to explain behaviours and issues that would otherwise go unnoticed.^20^

**Figure 1:**
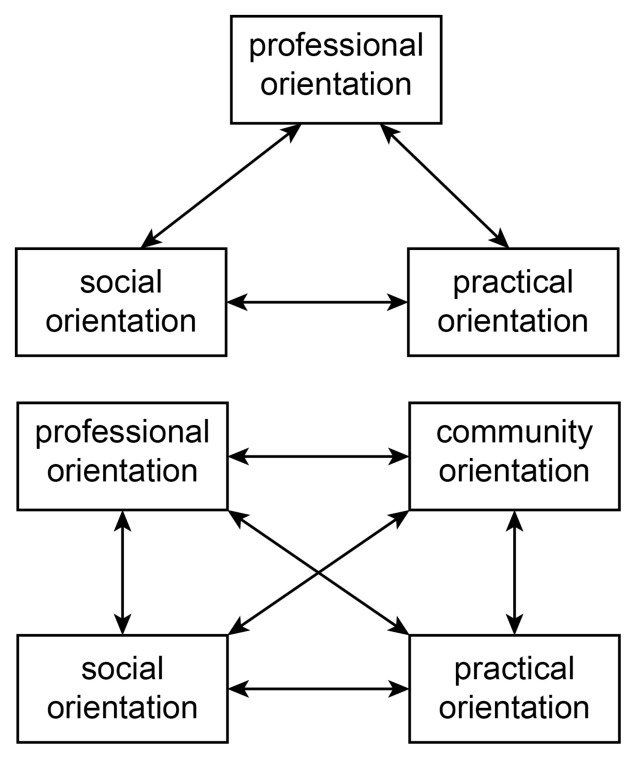
For most medical schools, orientation is based around three essential dimensions: social, professional or practical (as shown on the top).^1^ Orientation at the Northern Ontario School of Medicine adds a substantial dimension of community orientation to these interconnected dimensions (as shown on the bottom).

One of the key dimensions of these curricular concepts is the reflection of the beliefs and value systems, both collective (e.g. societal) and individual (e.g. instructor), that are present in an educational program. The pursuit of a social mission is a growing ideological stance taken by medical schools, often with a strong focus on the communities their trainees and graduates are meant to serve.^21^ The term “social mission” refers to a broad range of positions ranging from social responsibility (an intuitive commitment to the welfare of society), through social responsiveness (directing education, research and service activities towards explicitly identified health priorities), to social accountability (ongoing collaborations between medical schools and communities).^21^–^23^ Social accountability is often pursued through community-engaged activities, where community members are involved in the curriculum design, and participate in the evaluation of their education programs.^24^–^27^ Orientation to programs offered by a community-engaged medical school might therefore be expected to involve community dimensions alongside the social and practical dimensions of orientation to undergraduate medical training. We can represent “community” as an extension to the generic dimensions of medical school orientation (see Figure 1).^1^

Given the growing focus on the social mission of medical schools in Canada and the growing involvement of communities in medical education,^28^ what then might orientation to undergraduate medical education look like in these contexts? What hidden curricular messages might orientation activities be designed to send (even if they are not explicitly stated)? And, what other messages might emerge out of the design and implementation of orientation activities in and for these contexts? We focused on the undergraduate orientation activities at Canada’s newest medical school, the Northern Ontario School of Medicine (NOSM), as a case study of a medical education program in the context of an explicit and ambitious institutional social mission.

## Methods

### Study design

We undertook the broader study using a mixed-methods multi-source case study design.^29^ The case was bounded in terms of its locations (the one school and those communities visited during orientation) and its time (the three orientations at the end of August 2010, 2011, and 2012). The full case study was “instrumental”^30^ in that it was intended to illustrate broader issues of transition into medical school and designed as a single case with embedded units of analysis within the broad case study.^31^ We report the analysis of one of those units; an exploration of the students’ perspectives regarding orientation. We designed this component of the study to answer the research question: “what implicit messages did students experience in the orientation for undergraduate medical students at the Northern Ontario School of Medicine?”

### Case study context

NOSM’s mission is to be: “socially accountable to the needs and the diversity of the populations of Northern Ontario; actively involving Aboriginal, Francophone, remote, rural and underserviced communities; leading and conducting research activities that positively impact the health of those living in Northern communities; fostering a positive learning environment for learners, faculty, and staff; achieving an integrated, collaborative approach to education, learning, and programming; [and] increasing the number of physicians and health professionals with the leadership, knowledge and skills to practice in Northern Ontario.”^32^ This mission is reflected in the undergraduate program in many ways: community placements, community-focused cases and exemplars, and particular attention to the social determinants of health as applied to the peoples of Northern Ontario.

**Figure 2:**
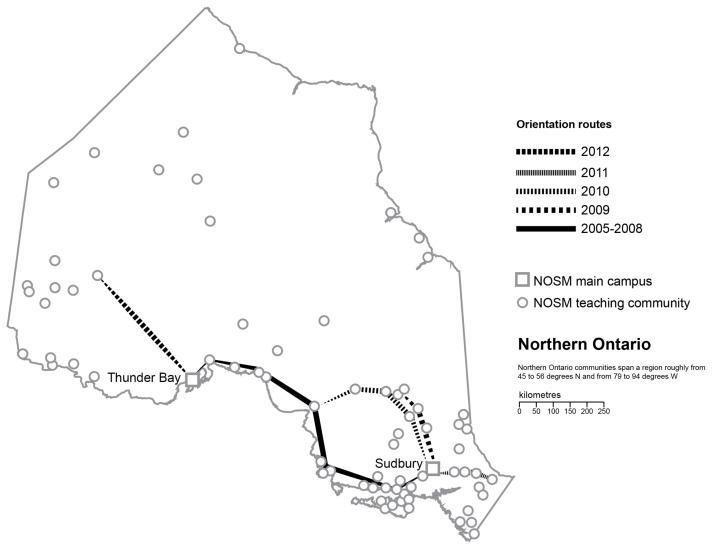
Northern Ontario showing the School’s teaching sites and the routes of the Orientation Week community visits from the 2005 Charter Class until the end of the study in 2012

NOSM admitted its first cohort of students in 2005.^33^ At the time of the study, the school was distributed across two campuses and more than 70 teaching sites over 800,000 square kilometres of the Canadian Shield (see Figure 2). The four-year MD program admitted 64 students per year - 36 in Sudbury (East campus) and 28 in Thunder Bay (West campus) - and it included 12 weeks of community placements in the first two years while the entire third year was made up of a longitudinal integrated clerkship in one of 15 mid-size communities.

**Figure 3:**
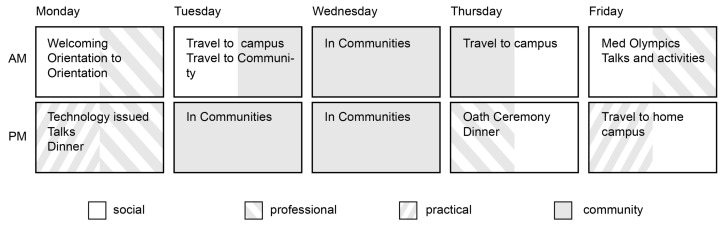
High level orientation week schedule for 2011

An Orientation Week (OW) had been run for the undergraduate medical program since the School opened in 2005. The OW format had undergone a number of changes over time to include a greater community component and to seek better alignment between its objectives and those of the undergraduate program and the School as a whole. A typical OW schedule at the time of the study is set out in Figure 3. Between 2005 and 2008, a significant part of the week consisted of a 1,000km bus trip from one campus to the other. From 2009 onwards, the format changed to focus on visits to clusters of communities. The objectives of OW at the time included: socializing as a class, building interpersonal connections between the two halves of the class at each campus, and developing an understanding of the diversity of different communities in the region.

### Participants

Incoming students in 2010, 2011, and 2012 (n=192) and all students already in the program in the 2010–2011 academic year (n=176) were invited to participate in surveys and focus groups (note the intake rose from 56 to 64 in 2010). We ran two first-year student focus groups - one in 2011 and another in 2012 - and one third-year focus group in 2011. Surveys were also sent to 29 faculty, managers, and staff in 2011 who had previously been involved in OW. Interviews with managers and staff responsible for organizing OW were conducted in 2011. Stakeholder surveys were sent (in 2011) to 15 physician and community leaders who had been involved in OW activities. There were no individual interviews conducted with students.

### Ethics

The Research Ethics Boards of Lakehead and Laurentian universities approved the study. Note that NOSM functions as the faculty of medicine for both universities and is obliged to submit studies involving its undergraduate students through both REBs.

### Data collection

We gathered data using participant surveys, focus groups, and key informant interviews. We developed the survey instrument from the pre-existing OW evaluation survey, adding free-text questions exploring participants’ experiences. Only the free-text comments were entered into the thematic analysis for this paper. Survey questions were the same for all three years of the study. The focus groups and key informant interviews employed the same semi-structured interview script. The script was developed iteratively in discussion amongst the study team members to elicit perceptions of OW without directly asking participants about the messages that they had taken from participating in OW. Given that we were unclear quite what kinds of messages and perceptions the study would uncover, we decided to take a naturalistic and open-ended approach that did not cue or otherwise bias participant responses by asking them directly about their perceptions of any apparent messages or values expressed in OW (see Appendix A). In doing so, we hoped that whatever comments they did make about perceived values and messages would reflect an authentic sense of what had intrigued or troubled them in OW.

Data from surveys were collected by the OW team rather than the study team, and then provided to the study team in a spreadsheet. Interviews and focus groups were audio recorded, transcribed using a commercial service, and manually checked and de-identified before sharing with the study team for analysis.

### Data analysis

Free text responses from the surveys and transcripts were analyzed as a single corpus by two independent coders (RHE and TD) drawing on constructivist grounded theory methods^34^ to identify recurring themes, including, but not limited to, hidden curriculum issues. We should be clear, however, that this was a case study conducted using grounded theory techniques at the analysis stage rather than a grounded theory study in its own right.^35^ To that end, the coding process involved independent line-by-line coding followed by iterative comparisons and consensus reviews by the coders to identify broader themes. The codes were iteratively checked against the data with weak or ambiguous topics either being condensed into stronger ones or deleted. Theoretical sensitivity (approaching data analysis from various vantage points)^36^ was obtained through continuous discussion with other members of the research team who compared outline findings and interpretations with the base data to check for representativeness and coders’ potential biases. Iterative changes were made to the framework to accommodate differing interpretations and observations.

## Results

There were 51 respondents (79.7% of the class) to surveys of incoming students in 2010, 64 (100%) in 2011, and 64 (100%) in 2012. There were 60 (34%) current student respondents (excluding first-year students) to the survey in 2010–2011, of which 20 had been involved in OW as student leaders and organizers. There were 26 staff survey respondents (90% of those invited), and 13 external stakeholder survey respondents (physician and community leaders - 87% of those invited). Two focus groups were held in 2011 with a mix of first- and second-year students (23 participants in total), and with third-year students at one of the School’s larger community sites (7 participants). Two focus groups were held in 2012 with a mix of first- and second-year students (18 participants in total). Six staff members who had been directly involved in planning and running OW were also interviewed.

On analysis, staff and stakeholder participants did not raise any particular concerns regarding the messages or positioning of OW that were intended but implicit within the program. Student responses on the other hand were rich with interpretations and concerns regarding their experiences with OW. Our analysis for this study therefore focused on student perspectives with staff and stakeholder responses acting as qualifiers and a normative background against which student comments could be contrasted.

We identified the following four overarching themes regarding the tacit and conflicted messaging around OW:

### 1: Community recruitment

Community-engagement was central to NOSM’s mission with training taking place in different community settings, many of which had chronic physician shortages. Approximately forty percent of the OW schedule was given over to community visits as a way of making incoming students aware of these issues. Not only did many communities welcome the students, many of them actively sought to recruit individual students to return and practice there once they had completed their training. One student observed that some communities were very direct in using visits for recruitment purposes: “… some of them were really explicit. [One community] gave us a proposal for [a substantial amount of money] if you come here once you’re finished.” It was evident that some students felt uncomfortable and conflicted by these recruitment strategies. For instance, one student shared the following: “It’s really good to see that the people of the North want physicians to stay in the North. But … I thought that as someone who didn’t necessarily want to go there that it was alienating and I was rebuffing them and their kindness.” However, there were also positive responses. For example, another student stated: “I liked how they started selling themselves.” Other students appreciated the needs of the communities they had visited, but thought that such active recruitment was inappropriate in orientation: “I think they would be wasting their time a bit if they didn’t at least put a plug in to let people know, hey, we are looking for doctors … but I also feel that it’s so early.” Although the social mission to address community health inequality was an explicit part both of the week and of the School’s mission, the community recruitment dimension of OW was not.

Cognitive dissonance between student expectations and the reality of community needs shifted the focus of OW from a celebration of their successes to establishing links between the School’s social mission and students’ first-hand experiences, which in turn had implications for their professional identity formation. As one senior student observed: “[OW] forms your sense of medical student identity, and your expectations, and what is expected of you in the coming years.” This was an important finding for a school with a community engagement and social accountability mandate. However, this “resetting of the compass” had the potential to backfire for those students who struggled with the ethical dimensions of misrepresenting their future plans (or the absence of plans) when pressured by community members. Following this study, incoming NOSM students were more robustly briefed about these kinds of encounters before they visited underserved communities in OW.

### 2: Influencing career choices

NOSM students are actively encouraged to consider practice in underserviced rural and remote areas. Nevertheless, not everyone who enters the NOSM MD program chooses a career as a rural family physician or a locus of practice in Northern Ontario,^37^ nor were they in any way obliged to do so. The mission of the School was to encourage and support students in making career choices that would benefit Northern Ontario but it could not, as an accredited Canadian medical school, place any obligations or restrictions on its students’ career choices. Some students entered into a reciprocal funding relationship with a community, where the student committed to return of service to that community in return for funding for their studies. However, these relationships were strictly between the student and the community. It was notable then that some students expressed a sense of being manipulated or pressured to respond positively to the perceived messages in OW around rural, remote, and primary care practice. For example: “As someone who wasn’t thinking … of family medicine when I came to the School it almost felt like you had to put your guard up and start pretending to be someone you weren’t.”

There was an apparent contradiction between respecting (and even celebrating) diversity and autonomy in students’ career choices, and seeking to advance the School’s mandate to address a chronic undersupply of rural family physicians in the region (an acknowledged issue for many schools with a social accountability mandate).^38^

### 3: Community representation

Students travelled significant distances from their campus cities to visit rural and remote communities during OW. Some students perceived this as sending mixed messages that larger communities were not as important as small and remote ones, at least from the School’s perspective. For some students this was not a matter of rejecting visits to smaller communities, but one of balance. For example, one student wanted to get to know the local hospital setting: “I really enjoyed the [smaller community] hospital visit … [but] if we had had that same hospital visit to the hospital here in [campus city], that might have been more of a useful experience.” Other students struggled to see the relevance of visiting communities other than their host campus community: “It was lovely to visit the different communities but I didn’t necessarily see the benefit of it in orienting ourselves to the School and the life we will have at the School.” The hidden curriculum here coalesced around a tension over what constituted a community for the purposes of orientation; communities as larger urban centres where students live and study (and from which the majority of the School’s students have been recruited) seemed less legitimate as exemplars in this context than those communities that were smaller, more rural, and remote. Students’ concerns over a lack of focus on their host campus communities were also practical. For instance, they wanted to know where the best places to eat and shop were in their communities (and had expected this to be a part of their orientation). The response to this issue was to raise the profile of the two campus communities in OW alongside the visits to rural and remote communities.

### 4: Challenges of bonding for a distributed class

A key institutional objective for OW was to forge each heterogeneous group of incoming individual students into a functional cohesive class. The main student concern was with regard to the dynamics of socializing a single class that was split across two campuses 1,000km apart. One student referred to the types of relationships that were formed during OW: “Am I investing in people that I’m going to see every day or am I investing in this friendship that I’m going to have to pursue long distance for the rest of the year?” Some students noted the importance of the sense of being one whole class: “[OW] provided an opportunity to meet these people and granted, we don’t see them often, but for me it helped a little bit more to feel like one class although for all intents and purposes we are two separate ones.” For some students there was too much of a focus on socialization: “It felt that it was a lot of time to be spending on bonding and icebreaking.” The more mature students tended to see this activity as trivial or lacking a professional focus. For example, one student reflected on the differences in some of the age gaps between students: “There were a lot of people in our class who were right out of undergrad … it’s not a criticism that young people in their 20s aren’t capable of professionalism but I do think it’s a matter of maturity.” The hidden curriculum here emerged from different participant perceptions of student-led activities that were intended to socialize the students to a shared class identity.

We also identified a number of more generic issues:

### Individual agency

Some students experienced a sense of diminished agency in OW, particularly those students who had had some kind of professional career before applying to medical school. Although all NOSM students were university graduates, many of them had pursued professional careers before turning to medicine (age on entry to medical school in 2012: national mean=23.1,^39^ NOSM mean 25.8^40^). For some, becoming a student again was a status challenge: “Calling us medical students creates a framework and a paradigm where we’re children.” A lack of autonomy in OW was also an issue for this group. For instance, one student argued that OW conflicted with the learning model of the School: “We are, for all intents and purposes, a self-directed school so why should orientation week be forced upon us?” Students entering directly from their first degree tended to be happier with the activities and the messages they took from them. The hidden curriculum here was associated with the expression of power and authority, both in terms of the agency afforded students to be truly self-directed, and in the labels and symbols presented. This sentiment tended to be experienced differently depending on apparent student maturity and life experience.

### School before program

Many students reported feeling better oriented to the School and its mission than to the practicalities of the MD program. This disparity reflected an ongoing debate within the School over what the focus of OW should be. It also became clear that the specific relevance of OW to the MD program had not been made clear or explicit to participating students. Moreover, although MD program leaders were involved in delivering OW, their focus tended to be on learning processes and team building rather than on the formal curriculum. Indeed, until very recently OW was not considered to be a part of the MD program *per se* and was organized by the Learner Affairs group rather than the MD program.

Although we have focused on tacit messaging and dissonance in student experiences in our analyses, we should note that most students reported having had a generally positive experience throughout OW. As an illustration of this favourable viewpoint, more than 95% of students responded “agree” or “strongly agree” to the survey question “Orientation Week has been helpful in my joining the School.” Nevertheless, the hidden curriculum issues we identified clearly nuanced the way students thought about themselves as medical students, and potentially the ways they approached their studies and their future relationships with the School.

## Discussion

Examining participants’ reflections on their orientation experiences is a different undertaking from researching discrete activities within a course context or the transitions within medical education.^19^ Even though the hidden curriculum is not necessarily negative and undesirable, using a hidden curriculum lens allowed us to observe points of confusion and dissonance arising from the messages students perceived in and around OW at NOSM. This allowed us to identify and then reflect on the embedded values (intentional or otherwise) in OW activities,^41^ how they were communicated, and the ways in which they impacted incoming students.

Although we used the concept of hidden curriculum as a research lens in its broader sense, we can reclassify some of the themes we identified using the more precise curricular concepts described earlier. For instance, the “bonding for a distributed class” and the “school before program” issues that arose from the organization of OW and the School were more strictly “hidden curriculum” factors. Whereas the “community representation” and “community recruitment” issues could be more properly categorized as part of the “informal curriculum,” and the “student agency and identity” and “influencing career choices” issues were part of the “rhetorical curriculum.”

We identified issues that were relatively context-specific (at least to schools with a more explicit social mission), such as the tension between institutional social accountability and personal career choices, as well as those that are common to more heterogeneous medical schools, such as tensions between institutional and student responsibility for orientation.^1^ Many of these issues were experienced asymmetrically across the class; some students perceived problems and concerns with certain aspects of OW, while others did not. There are parallels in this with studies of professional identity formation, particularly those that have shown how some students’ current identities and values may be more at odds with their chosen profession than those of their peers.^42^ However, although participants did discuss aspects of OW that were misaligned with their particular sense of self or their expectations, professional identity formation was not discussed explicitly. This is attributable, in part, to our deliberately naturalistic stance, in that we did not explicitly explore professional identity formation with our participants, not least because we did not expect a sense of professional identity be particularly explicit or well-developed in individuals who had been in medical school for just a few days.

Nevertheless, it became apparent that the sense of conflict or discomfort some participants experienced was exacerbated by their perceptions of the social pressures of OW. While the goal (and, for many, the result) of these experiences was to engender a greater commitment to the mission of the School as it pertained to their training and career choices, there were those for whom this proved counterproductive in shaping their relationship with the School, both in the moment and subsequently. Although NOSM’s mission is quite explicit (certainly more so than most schools in North America), its students are exposed to a wide range of professional career options during their studies, and its graduates have matched to general specialty and sub-specialty residencies as well as to family medicine programs. That some students perceived a level of coercion to choose rural family medicine careers over the alternatives warrants further investigation.

Although NOSM differs from Canada’s other 16 medical schools in several ways, this study does have broader implications for Canadian medical education, and potentially for programs elsewhere in the world. Social accountability has become an accreditation standard for undergraduate medical education programs in Canada, which means that, even for schools with more modest social missions, it is important that they understand the dynamics of how an institutional mission and its educational activities can and do intersect. Given the values-laden nature of a social mission, it should be acknowledged that some, otherwise well-intentioned, ways of engaging students may be experienced as manipulative or even coercive. However, medicine does not exist in a social, cultural, or political vacuum, and helping students to confront the realities of the social mission at the start of their training may be a more effective way of orienting students to their roles and social responsibilities. More research is needed to explore these issues, but we have in this study set the groundwork for further investigation of the development of social awareness and moral responsibility at the start of training rather than trying to add it later in the arc of professional identity formation.

We acknowledge a number of limitations to this study. First, given that this was a case study, its intrinsically ideographic nature could mean that it is of limited generalizability.^43^ However, we would position this work within Flyvbjerg’s concept of a both “critical” and a “paradigmatic” case^44^ in that NOSM has arguably the most explicit commitment to a social mission of any of Canada’s 17 medical schools. This study also reflects Flyvbjerg’s other utility criteria for value in case study research. First, there is substantial value in concrete, context-dependent knowledge in complex situations such as these. Next, generalizability here is a matter of the case defining a perimeter rather than an exemplar for Canadian medical education. Finally, we did not seek to verify students’ understanding of the NOSM mission or how it was received, rather we demonstrated several ways in which perceptions and impacts could differ from the institutional ideal.^44^

We also acknowledge that NOSM is not a typical Canadian medical school and that its OW does not reflect common practice. However, having a social mission is now an accreditation standard in Canada^45^ and it is a key part of the Future of Medical Education in Canada initiative.^46^ Another factor that we did not substantially explore in this study was the risk management side of orientation. Medical schools’ risk liability is a growing concern in terms of the reputational damage associated with student misadventures during orientation programs.^47^ We would therefore argue that this study is particularly relevant as a paradigmatic case, both in terms of orientation issues in general and more specifically around the expression of a social mission in undergraduate medical education.

We can reflect on the quality of the study using the framework from Morse et al.^48^ We addressed methodological coherence by following an inductive approach of seeking unprompted and therefore naturalistic perspectives on the experiences of participants in OW. We did not explicitly ask about hidden messages or the hidden curriculum. Furthermore, our sample was appropriate in that we gathered data from all constituent groups in OW, our analyses moved back and forth between confirming known factors in OW and discovering new and variant ones, and we iteratively developed and tested explanatory theories to ground our findings. However, there was a possibility of significant bias given that the majority of the research team were also senior leaders in the School at the time of the study, several of them with direct or indirect responsibility for OW. The two coders had the greatest distance from OW (RHE and TD) and they took a deliberately critical stance to be able to better identify and potentially counter biases arising from the perspectives of the rest of study team. Nevertheless, we were faced with issues that were not always comfortable and that were not easily addressed or even fully understood within the framing of the study. We took this sense of discomfort as an indicator of the efficacy of this approach, even though there was almost certainly more to discover.

Given the voluntary nature of participation, it is possible that those who elected to contribute to the study had unrepresentative opinions and concerns regarding their experiences of OW and the School in general. However, given that our findings are about the asymmetries of experience within a class and the diverse ways that individuals respond to experiences linked to a school’s social mission, these “black swans” are a critical part of a paradigmatic case study^44^ and as such this would be a strength of the study rather than a weakness.

Finally, the conceptual basis for this study has emerged in a relatively unoccupied space between the literatures on transitions, hidden curriculum, and professional identity formation, the latter of which emerged from our analyses rather than from our original conceptualization of the study’s theoretical underpinnings. We have suggested conceptual frames for advancing scholarship in this area but in doing so we must acknowledge the specificity of NOSM’s context and mission (as well as the idiographic nature of this study), and as such, there are limitations to the generalization of our findings. We look to other schools to explore orientation in their contexts, cultures, and systems of medical education to advance scholarship in this largely unexplored area.

### Conclusion

The limited attention paid to orientation to medical school in the literature may reflect a general sense that it is a somewhat benign and uncomplicated point of transition. We have shown that this is not the case, at least it was not for NOSM at the time of the study, and we have identified broader issues of concern to medical education scholars. Orientation is rich in hidden curriculum messages that have the potential to influence students’ professional identity formation, to reset their compass as it were, particularly in the context of a school with an explicit social mission. Given that NOSM actively recruits students who can be change agents as well as competent physicians, there should perhaps be no surprise that they take a critical stance with respect to their experiences. As scholars, we continue to evaluate and develop evidence-based orientation practices to prepare students for their medical education and to advance the mission and vision of their schools.

## Appendix A Interview Questions

The interviews, focus groups, and staff and stakeholder surveys were based on the same set of questions:

1. Which orientation weeks were you involved in and in what capacity?
2. Can you describe your involvement with the School’s orientation process?
3. What is your overall impression of the School’s orientation process?
4. What are your most vivid memories of the School’s orientation process?
5. What were the most important or useful parts of the School’s orientation process for you?
6. What were the least important or useful parts of the School’s orientation process for you?
7. How do you think the School’s orientation process should be improved?
8. Do you have any other comments about the School’s orientation?
